# The Role of Tumor-Derived Vesicles in the Regulation of Antitumor Immunity

**DOI:** 10.32607/20758251-2019-11-4-33-41

**Published:** 2019

**Authors:** V. M. Ukrainskaya, Y. P. Rubtsov, V. D. Knorre, M. A. Maschan, A. G. Gabibov, A. V. Stepanov

**Affiliations:** M.M. Shemyakin and Yu.A. Ovchinnikov Institute of Bioorganic Chemistry of the Russian Academy of Sciences, Moscow, 117997 Russia; Dmitry Rogachev National Medical Research Center of Pediatric Hematology, Oncology and Immunology, Moscow, 117997 Russia

**Keywords:** vesicles, exosomes, immune response, CD4+/CD8+ T cells, tumor microenvironment

## Abstract

In this article, we present a comprehensive, updated, and elucidative review of the current knowledge on the function played by tumor-derived vesicles (TDVs) in the crosstalk between tumor and immune cells. Characterization of the structure, biogenesis, and the major functions of TDVs is reported. The review focuses on particular ways of suppression or activation of CD4^+^/CD8^+^ T cells by tumor-derived vesicles. Tumor-derived vesicles play an important role in the suppression of antitumor immunity. During the last 15 years, vesicle research has elucidated and improved our knowledge about the role of the vesicles in intercellular communication. Nevertheless, there are still blinds spots concerning vesicle heterogeneity and isolation methods, their uptake by target cells, and the role of mRNA in T-cell transformation or suppression. Along with the substantial progress in understanding of the role of tumor-derived vesicles in intercellular communication, novel antitumor therapy strategies based on vesicle inhibition in a tumor microenvironment are likely to appear very soon.

## INTRODUCTION

New data on the origin, composition, and influence of extracellular vesicles (EVs) on cells have significantly changed their functions and significance. While being earlier regarded as "cellular debris," EVs have become a new means of intercellular communication. It turns out that these structures (typically the intracellular ones) are actively involved in the regulation of the immune response, as well as other processes that require intercellular communication [1, 2]. The observation that EVs can modulate the phenotype and function of target cells at the genetic and epigenetic levels by transferring genetic material (usually different types of RNA) was an extremely important step in the "biography" of vesicles [1]. The secretion of extracellular vesicles by both normal and tumor cells makes them an important component of the tumor microenvironment. It should be emphasized that tumor cells secrete more vesicles in comparison to the normal cells of the surrounding tissue, which can be attributed to the fact that they proliferate rapidly under constant stress conditions [3-5]. Many factors can influence EV production by cells. Thus, a low pH in the tumor microenvironment is known to be important for maintaining the stability of the lipid/cholesterol composition of vesicles [6]. Changes in the number and composition of vesicles correlate well with the severity and prognosis of many diseases. This fact allows one to use EVs as a non-invasive diagnostic tool [7].

Being a component of the cellular environment, vesicles are apparently involved in cell differentiation, division, and maintenance/alteration of the cell phenotype both in normal cells and in various pathologies, including cancer [2]. Although tumor-derived vesicles (TDVs) suppress the immune system and contribute to tumor development, they simultaneously contain tumor antigens. This property of vesicles could potentially be used in immunotherapy for eliciting an antitumor immune response [8].

This review discusses how tumor-derived vesicles are involved in the immune response regulation and affect the function of CD4^+^/CD8^+^ T cells in the context of a tumor microenvironment.

## 1. CHARACTERISTICS OF EVs

The term "extracellular vesicles" is used to describe spherical cellular structures (30–1000 nm in size) enclosed in a lipid bilayer. According to their average size and biochemical profile (a combination of their components), vesicles are classified into different types. The difficulties associated with obtaining pure vesicles and physical isolation of their individual types make accurate vesicle classification rather challenging [9]. Extracellular vesicles can theoretically be classified according to their size or origin [9, 10].

1) Exosomes are structures of endosomal origin (30–150 nm in size) that carry characteristic markers belonging to the tetraspanin (CD9, CD63, CD81) and chaperone (Hsp70, Hsp90) families.

2) Microvesicles are cytoplasmic particles that actually are budded cell membrane fragments. Their size ranges from 100 to 1000 nm.

3) Apoptotic bodies are large (typically described as 1000–5000 nm) fragments of cells being formed during apoptosis.

In this study, we provide a detailed description of exosomes and microvesicles, which are further referred to using the general term "vesicles" (or EVs).

**1.1. Vesicular composition**

**Fig. 1:**
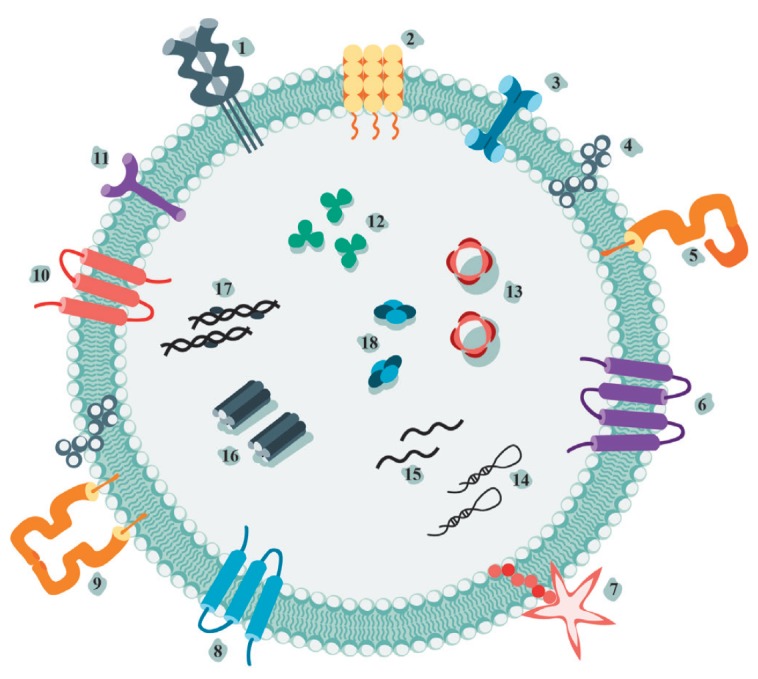
Schematic representation of a typical vesicle with the most common components. Adhesion molecules (integrins (11), tetraspanins (6, 7, 8, 10)); signal transduction molecules (syntetins, annexin V(3)); major histocompatibility complex molecules (MHC class II (5) and MHC class I (9)); cytoskeletal proteins (actin, myosin (16)); heat shock proteins (12); lipids (ceramide (4)); proteins responsible for vesicle biogenesis (13); and proteins of metabolism (GAPDH(18)). 1 – FasL; 2 – ICAM-1; 3 – annexin V; 4 – ceramide; 5 – MHC I class; 6 – tetraspanin CD81; 7 – CD80; 8 – tetraspanin CD9; 9 – MHC class II; 10 – tetraspanin CD63; 11 – integrin; 12 – heat shock proteins; 13 – AUX/Alix; 14 – microRNA; 15 – mRNA; 16 – actin, myosin; 17 – DNA with histones; 18 – GAPDH (glyceraldehyde-3-phosphate dehydrogenase)

EVs contain a set of proteins that are characteristic markers of the cell line they have been derived from [11]. For example, tubulin proteins (TUBB4B and TUBA1C) are found in vesicles derived from lung cancer cells [12], while CD20 is present in B-cell lymphoma-derived vesicles [13]. The protein and lipid compositions of vesicles (shown in more detail in *Fig. 1*) has been studied in an attempt to understand the entire range of effects exerted by tumor-derived vesicles on immune cells [14, 15, 16]. Any tumor-derived vesicles are characterized by the presence of tetraspanins (CD63, CD81, and CD9), whose amount may vary depending on the tumor type and stage of progression [17]. Major histocompatibility complex (MHC) class I and II molecules can also be found on the vesicle membrane (*Fig. 1*), which is especially important for EVs secreted by antigen-presenting cells (APCs) [18, 19]. Along with proteins and lipids, vesicles can also contain genetic material (DNA [16, 20, 21], ribosomal RNA, messenger RNA, as well as microRNA and other non-coding RNAs). The mechanism via which nucleic acids are loaded into EVs has not been fully elucidated, but certain "barcodes" (short microRNA and RNA sequences specific to RNAs isolated from vesicles) have been detected [20, 21, 22]. Databases on the molecular composition of vesicles, such as EVpedia [23], Vesiclepedia [24], and Exocarta [25], provide a thorough description of the protein and lipid components found in different types of EVs.

**1.2. Biogenesis of vesicles and how they crosstalk with target cells**

**Fig. 2:**
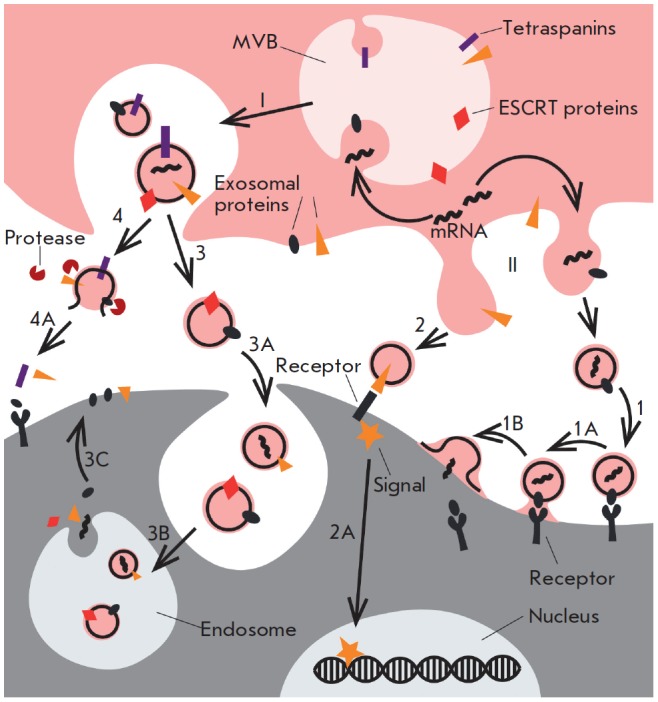
Biogenesis of EVs and their uptake by the target cells. The EV biogenesis pathways (I, II). I – EV biogenesis in MVBs via the ESCRT-dependent/ESCRT-independent pathways: fusion of MVBs with the plasma membrane; II – EV formation by direct budding from the plasma membrane into the extracellular space. The interaction between the secreted membrane vesicles and recipient cells (1, 2, 3, 4). 1, 2 – Binding of secreted vesicles to the surface of a recipient cell involves interactions between exosomal ligands and cellular receptors, fusion with the plasma membrane (1A) and release of vesicle components into the cytoplasm (1B); 2A – activation of signal pathways; 3A – vesicle endocytosis; 3B – fusion of the endocytosed exosomes with the limiting membrane of the endosome; 3C – incorporation of exosome membrane proteins into the endosome membrane, which could then be recycled to the cell surface; 4 – exosome degradation by extracellular proteases; 4A – interactions between exosomal ligands and cellular receptors

Exosomes and microvesicles form in the cell via different pathways *(Fig. 2)* [26]. Microvesicle budding from the cell membrane is mediated by cytoskeletal proteins (actin, myosin, etc.), neutral sphingomyelinase N-SMase that is involved in ceramide formation, as well as ARF6 (ADP-ribosylation factor 6) and phospholipase PLD2 [27, 28]. The endosomal sorting complex (ESCRT), which sends ubiquitinated proteins into multivesicular bodies (MVBs), is involved in the formation of both microvesicles and exosomes [26, 29]. The ESCRT consists of four protein components (ESCRT-0, -I, -II and -III), which consecutively bind proteins to form intracellular vesicles [7]. In turn, the accessory proteins syndecan–syntetin–(ALG-2-interacting protein X) trigger exosome budding from the membrane into MVBs [30]. According to the available data, formation of glycolipoprotein microdomains (lipid rafts) containing neutral sphingomyelinase (N-SMase) is an alternative pathway of EV biogenesis. Ceramide synthesis by sphingomyelinase and its accumulation in the membrane cause raft merging and formation of an exosome in the MVB cavity [31]. The endosomes inside MVBs are released into the extracellular space via fusion between the cell membrane and MVBs; this process is regulated by GTPases belonging to the Rab and Ras families, as well as the SNAPE protein (soluble N-ethylmaleimide-sensitive factor attachment protein receptor) [21, 32].

The existence of several pathways of EV biosynthesis has been confirmed in many studies [33]. Hence, formation of MVBs was observed in cells lacking ESCRT proteins, although the budded vesicles had a nonconventional composition and morphology [31]. The inhibition or knockout of sphingomyelinase N-SMase reduces vesicle secretion by cells and suppresses the metastatic spread and angiogenesis in tumor [34]. However, there is doubt about a spontaneous formation of EVs via the ESCRT-independent pathway, since it has been proved that lipid rafts are not needed for exosome formation [35]. Although the pathways of EV biogenesis are theoretically subdivided into an ESCRT-dependent and ESCRT-independent one, formation of a given population may depend on each pathway to a different extent [9, 36].

EVs can possess various functions in a tumor microenvironment, but almost all of these functions are implemented when a vesicle interact with the target cell [2]. There are at least four different ways through which EVs carry protein molecules to the cell surface or deliver them inside cells *(Fig. 2)*.

• contact between specific vesicle molecules exposed at the exterior of the membrane and the receptors of recipient cells, making activation of the intracellular signaling cascades possible [2, 19];

• cleavage of surface vesicle proteins by extracellular proteases, followed by crosstalk between vesicular proteins and membrane receptors;

• fusion of the vesicular and cell membranes, followed by either release of the intravesicular content into the cytoplasm or endosome formation [26]; and

• phagocytosis and the uptake of an entire vesicle by a recipient cell [5, 21, 37].

The crosstalk between vesicular tetraspanins, proteoglycans, lectins, and integrins and membrane receptors of the recipient cell triggers vesicle penetration of the cell, which can be blocked by an antibody specific to a given vesicle protein. For example, treatment of vesicles with the anti-CD81 or anti-CD9 antibody or blockage of proteoglycans by heparin sulfate reduces vesicle adhesion to the recipient cell. Vesicle endocytosis can also be blocked using cytochalasin B or latrunculin A, which inhibits cytoskeletal components (actin and fibronectin) [2]. Secretion of vesicles and their endocytosis are processes that have mostly been studied in vitro thus far. These processes need to be studied in vivo as well to elucidate the physiological role of their effect on surrounding cells.

Hence, a conclusion can be drawn that the diversity of EVs and their protein composition, as well as the multiple variants of crosstalk between EVs and target cells, suggest that EVs are a multifunctional component of any physiological or pathophysiological process. Vesicles can have various functions depending on their cellular origin: from regulating the immune responses and suppressing tumor invasion to being involved in intercellular communication. Studying the question of how these nano-sized structures in the cellular environment exhibit diametrically opposed effects could allow one to use vesicles as targets for anti-tumor therapy or as "liquid biopsy" for diagnosing tumor invasion [38].

## 2. TUMOR MICROENVIRONMENT: THE IMMUNE RESPONSE AND THE ROLE OF TUMOR-DERIVED VESICLES

Different cell populations forming the stroma (fibroblasts) and the immune environment (tumor-infiltrating lymphocytes, macrophages, myeloid-derived suppressor cells, etc.) are present in a tumor microenvironment. The complex of immune reactions is mediated by T cells, which not only trigger and stimulate (CD4^+^ T cells, T-helper cells (Th)) or regulate (regulatory T cells (Treg)) the immune response, but also destroy infected or tumor cells (CD8^+^ killer T cells, cytotoxic T cells). The eradication of tumor cells and memory cell formation is a reasonable result of the T-cell immune response [8]. In turn, tumor cells find various ways to "evade" the immune response. As suggested by the available data, releasing tumor-derived vesicles is one such way.

**Fig. 3:**
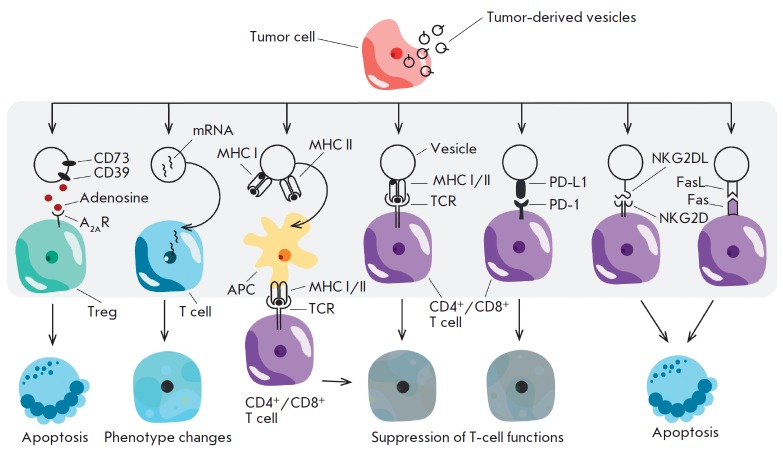
Effects of tumor-derived vesicle on immune cells. This image shows how tumor-derived vesicles can activate or suppress different populations of immune cells via various mechanisms

The following mechanisms via which tumor-derived vesicles can contribute to tumor evasion of immunosurveillance have been identified (*Fig. 3*):

(1) initiating apoptosis in cytotoxic CD8^+^ T cells [39];

(2) shifting the phenotype of CD4^+^ T cells towards Tregs [40, 41];

(3) transduction of tumor-associated antigen by vesicles and its presentation to cells other than professional APCs or to immature APCs, thus causing T-cell anergy in the absence of costimulatory signaling [42, 43, 44];

(4) regulated suppression of the T-cell immune response, which depends on various mechanisms [45];

(5) macrophage re-programming to an M2 phenotype (supporting the tumors) [39, 46, 47]; and

(6) slowing down the proliferation of NK cells [40].

Below, we discuss the pathways through which tumor-derived vesicles can affect CD4^+^/CD8^+^ T cells *(Fig. 3)* in more detail.

## 3. THE NEGATIVE EFFECTS OF THE CROSSTALK BETWEEN EVS AND THE SURFACE RECEPTORS OF TARGET CELLS

**3.1. Vesicles induce the apoptosis of CD8^+^ cytotoxic T cells**

The release of EVs carrying apoptosis activation factors by tumor cells is considered to be one of the immunosuppression mechanisms [48, 49]. When incubated with Fas^+^ T cells, EVs carrying the highly active membrane protein FasL contribute to cytochrome *c *release into the cytosol, loss of the mitochondrial membrane potential, caspase activation, and DNA fragmentation in T-cell chromatin [48, 50, 51]. The coexpression of FasL and TRAIL on the surface of secreted tumor-derived vesicles also induces apoptosis in CD8^+^ T cells [52]. Vesicles released by tumor cells induce apoptosis of Th1 cells via the galectin-9/Tim-3 crosstalk [53]. In turn, vesicles derived from normal cells (fibroblasts or dendritic cells) do not induce the apoptosis of activated cytotoxic CD8^+^ T cells [54].

It has been experimentally proved that reduced expression of the costimulatory molecule CD3ζ can be observed in T cells in a tumor microenvironment, which results in T-cell anergia and correlates with a decreased release of cytokines such as IL-2, IL-7, and IL-15. Vesicles containing FasL^+^ can exhibit this capacity: by interacting with Fas+ lymphocytes, they reduce the number of CD3ζ and JAK3 (Janus kinase 3, tyrosine-specific protein kinase 3) molecules in T cells that have undergone primary activation and facilitate the transition of cells to their apoptotic state [55].

The NKG2D/NKG2DL system also plays an important role in immune cell survival [56, 57]. The NKG2D receptor (Natural Killer Group 2D, a natural killer cell receptor) resides on the membrane of NK cells and CD8^+^ T cells [58]. MHC class I-like molecules and UL16-binding proteins act as ligands (NKG2DL) of this receptor; they are poorly represented on the surface of normal non-stressed cells. The emergence of these molecules on the membrane is activated by cellular stress (a viral infection or malignant transformation) [59]. Tumor-derived vesicles expressing various NKG2DLs bind NKG2D on the surface of NK and CD8^+^ T cells, thus blunting the cytotoxic function of T cells [60-62].

**3.2. Suppression of T-cell activation via PD-L1/PD-1 crosstalk**

The physiological role of the PD-1 (Programmed death-1) immune receptor is to regulate excessive activation of lymphocytes. When interacting with its ligand (PD-L1), the PD-1 receptor transduces a negative signal inside the T cells, which inhibits their proliferation and increases apoptosis. Recent studies have demonstrated that PD-L1 resides on tumor-derived vesicles, allowing them to suppress T-cell activation [47, 63, 64]. In particular, melanoma cells secrete PD-L1+ EVs in which the PD-L1 level is directly proportional to the level of IFN-γ secreted by lymphocytes [65]. In vivo and in vitro studies showed that hepatocellular carcinoma cells also release PD-L1+ vesicles, which inhibit CD4^+^ and CD8^+^ T cells via the PD-L1/PD-1 crosstalk [66, 67]. When PD-L1-positive vesicles interacted with T cells, the suppression effect was eliminated by pre-incubation with the anti-PD-L1 antibody, which blocked PD-L1 on the vesicles [67].

**3.3. Release of immunosupressive adenosine**

Adenosine is known to be an immunosuppressive factor [40]. It interacts with one of the isoforms of the adenosine receptor (A_2A_R) expressed on the T-cell surface and increases the cAMP level in CD4^+^ T cells, thus suppressing their activation [40]. ATP hydrolysis to adenosine is catalyzed by CD39 (an ATP hydrolase converting ATP to 5’-AMP) and CD73 (a 5’-nuclease converting 5’-AMP to adenosine) [68].

Tumor-derived vesicles often carry both of these enzymes (i.e., they are in the CD73^+^CD39^+^ status), which has a negative impact on T cells in a tumor microenvironment [69]. CD73^+^CD39^+^ vesicles induce adenosine secretion; they also activate inosine biosynthesis upon longer contact with cells [70]. Inosine maintains long-term activation of the A_2A_R receptor on Tregs, which in turn has a strong suppressive effect on CD4^+^ T cells [71]. It was found that this indirect signal from tumor-derived vesicles is much stronger than that from the cells, as evidenced by the significant contribution of EVs to intercellular communication [72].

## 4. CHANGES IN CELL BEHAVIOR CAUSED BY ENDOCYTOSIS OF VESICULAR COMPONENTS

**4.1. Vesicular RNA modulates T-cell functions**

Vesicles contain various types of RNA; mRNA and microRNA being the most abundant and diverse RNA types. 18S and 28S ribosomal RNA and DNA are less abundant. The ExoCarta database based on the results of 286 studies contains approximately 6,000 characterized microRNAs and mRNAs isolated from EVs [73]. The horizontal transfer of mRNA from a vesicle to the target cell may affect the transcription level of some genes which are involved in such processes as suppression/amplification of T-cell functions (in particular, for cells responsible for the production and secretion of proinflammatory cytokines and other biologically active molecules).

The tumor and its microenvironment are involved in the induction of active Tregs and contribute to the conversion of CD4^+^CD25^-^ naïve T cells into CD4^+^CD25^+^ Treg cells. EVs can also induce the conversion of CD4^+^CD25^-^ T cells into the CD4^+^CD25^+^Foxp3^+^ phenotype of Treg cells. This conversion of naïve T cells into Tregs requires phosphorylation and coactivation of the transcription factors Smad2/3 (Similar to Mothers Against Decapentaplegic 2/3) and STAT3 (Signal Transducer and Activator of Transcription 3) [74, 75]. The enhanced intensity of Treg formation leads to an imbalance in the proportions of immune cells in a tumor microenvironment, thus inducing the TGFβ- dependent mechanism of apoptosis of effector T cells. In turn, CD4^+^CD25^+^Foxp3^+^ Treg cells can also release EVs, which suppress the proliferation of type 1 T helper cells (Th1) and CD8^+^ T cells and reduce IFN-γ secretion by these cells [74, 75, 76].

The verified increase in the number of Tregs in tumor is accompanied by a reduction of the number of differentiated Th1- and Th17-lymphocytes, leading to a Treg/Th imbalance [77, 78]. In the presence of this imbalance, specific microRNAs miR-29a-3p and miR- 21-5p of vesicular origin are detected in target cells [79]. As they accumulate in the cells, these microRNAs can affect various signaling pathways associated with the suppression of T-cell activation. Activation of the MAPK1 (mitogen-activated protein kinase) signaling cascade, the STAT3/JAK1 pathway, and other signaling pathways in CD4^+^ T cells mediated by vesicular microRNA disturbs the cytokine profile of Th and Th17 cells and changes the lymphocyte phenotype of Tregs [80, 81].

The effect of vesicular mRNA on T-cell functions directly depends on whether T cells are naïve or activated. Tumor-derived vesicles were found to significantly increase the expression of genes having a verified immunity-regulating function in activated CD4^+^ T cells, while in naïve cells, gene expression slightly increased only for FAS1, IL-10, and PTGS2, while decreasing for DPP4, CD40LG, and NT5E [82].

**4.2. T-cell activation/suppression by EVs carrying antigen-presenting complexes on their surface**

Antigen-presenting cells (APCs) are also capable of releasing vesicles. Moreover, these vesicles carry MHC II (major histocompatibility complex class II) and can indirectly stimulate activated CD8^+^ T cells but not naïve ones [51]. This process is regulated by the crosstalk between the T-cell receptor (TCR) on the T-cell membrane and the MHC–peptide complex in the presence of additional costimulatory signaling from the CD28/B7 molecules or LFA-1/ICAM-1 adhesion molecules presented on the vesicle surface. The crosstalk between TCR and MHC in the absence of costimulatory signaling is known to cause T-cell anergy (i.e, makes cells unable to divide and secrete cytokines in response to the stimulation of the T-cell receptor) [83, 84].

It has been found that vesicles derived from melanoma cells are also able to transfer MHC I from tumor cells to APCs, thus changing the expression profile of receptors on the APC surface. EV-derived cytokines and mRNA potentially have an immunosuppressive effect on APCs and decrease the amount of MHC I and CD40 molecules on the cell surface [83]. An APC phenotype shifting toward an immunosuppressive one reduces the probability of stimulation of cytotoxic T cells, which may be the mechanism via which tumor cells "evade" the immune response [83, 84].

## 5. CONCLUSIONS

To sum up, it is worth mentioning that there is evidence pointing to the fact that tumor-derived extracellular vesicles may be a crucial factor in the formation of an immunesuppressive microenvironment. The negative effect on the immunity can be regulated by receptor-mediated crosstalk between the target cells and EVs, causing T-cell anergy or apoptosis. EVs and their contents can be uptaken by target cells, also leading to transduction of the immunosuppressive signal. The vesicular activity can be one of the reasons behind the treatment resistance and the phenotypic changes in tumor cells induced by chemo- and radiotherapy. Since the effect of EVs on immune cells, and T cells in particular, has been studied insufficiently, a relevant fundamental and practical problem is to characterize EVs and identify the molecular mechanisms underlying their binding and biological effect.

## Abbreviations

CD: cluster of differentiation
EVs: extracellular vesicles
Hsp: heat shock proteins
TUBB4B, TUBA1C: tubulin beta
MHC: major histocompatibility complex
APC: antigen-presenting cell
N-SMase: sphingomyelin phosphodiesterase
ARF6: ADP-ribosylation factor 6
ESCRT: the endosomal sorting complexes required for transport
MVBs: multivesicular bodies
PLD2: phospholipase D2; Th – T helper
Treg: regulatory T cells
NK: natural killer
TRAIL: tumor necrosis factor ligand superfamily member 10
IL: interleukin
NKG2D: natural killer group 2 member D
PD-1: programmed cell death 1
A2AR: adenosine A2A receptor
cAMP: cyclic adenosine monophosphate
STAT: signal transducers and activators of transcription
TGFβ: transforming growth factor beta
IFN-γ: interferon gamma; JAK – janus kinase
MAPK: mitogen-activated protein kinase
LFA: lymphocyte function-associated antigen 1
ICAM: intercellular adhesion molecule 1
TCR: T-cell receptor
